# Editorial: Identification and Characterization of Novel Antigens of Malarial Parasites

**DOI:** 10.3389/fcimb.2022.921027

**Published:** 2022-05-16

**Authors:** Md Atique Ahmed, Feng Lu, Yang Cheng, Jin-Hee Han

**Affiliations:** ^1^ Malaria Division, Indian Council of Medical Research (ICMR)-Regional Medical Research Centre, Dibrugarh, Assam, India; ^2^ Department of Pathogen Biology and Immunology, School of Medicine, Yangzhou University, Yangzhou, China; ^3^ Department of Public Health and Preventive Medicine, Wuxi School of Medicine, Jiangnan University, Wuxi, China; ^4^ Department of Medical Environmental Biology and Tropical Medicine, School of Medicine, Kangwon National University, Chuncheon, South Korea

**Keywords:** malaria, plasmodium, protein, antigen, antibody, Plasmodium falciparum, Plasmodium vivax, Plasmodium knowlesi

Malaria is a life-threatening infectious disease caused by *Plasmodium* species. There are about 250 *Plasmodium* species discovered in the world. Amongst them, only five species are known to infect humans. Globally, there were 241 million malaria infections and 627,000 deaths in 2020. According to the World Health Organization (WHO), malaria incidences have significantly increased as a result of the COVID-19 pandemic, which has caused a disruption in malaria prevention, diagnosis, and treatment (World Health Organization, 2022), reminding us that we need to consistently control malaria. Recently, the WHO urgently approved the RTS,S/AS01 vaccine termed Mosquirix for the prevention of malaria; however, actual vaccination efficacy was predicted to be only 30 percent (RTS,S Clinical Trials Partnership, 2015). Because of the complicated life cycle of malaria parasites, the finding of vaccine candidates has proven to be extremely difficult. *Plasmodium* parasites life cycle are shared in two hosts: mosquitoes serve as the definitive host and humans serve as the intermediate host. On the human side, the parasite undergoes a rapid transformation from the sporozoite to the merozoite stage. Despite the efforts of numerous researchers to uncover novel malaria vaccine targets, there is still huge gap in knowledge of malaria antigens at the molecular level. A single malaria merozoite encodes about 5,000–8,000 genes that are necessary for its survival, and these proteins could be potential vaccine or therapeutics target (Gardner et al., 2002). To date, we only have information on around 0.6-1.0% blood stage malaria antigens at the molecular level (Kanjee et al., 2018), which is a major limitation when deriving vaccine strategies to prevent malaria. We had better improve the knowledge of essential human malaria protein properties to overcome this big limitation.

Furthermore, there are at least 3 non-human primate *Plasmodium* species (*P. knowlesi*, *P. cynomolgi* and *P. brasilianum*) that potentially infect humans by chance or naturally (Ta et al., 2014; Ahmed and Cox-Singh, 2015; Lalremruata et al., 2015). In 2004, *Plasmodium knowlesi* was recognized as a zoonotic malaria that infects humans and old-world monkeys in South-East Asia (Cox-Singh and Singh, 2008). When we consider that humans and monkeys share more than 90 percent of their genomes, the zoonotic malaria instances are not surprising. The number of clinical case reports of zoonotic malaria cases in the world has increased in recent years, with the majority of cases occurring in South America and Southeast Asia, where non-human primates act as primary host (Lalremruata et al., 2015; Brasil et al., 2017; Imwong et al., 2019; Putaporntip et al., 2021). Thus, we must have a comprehensive understanding of human and non-human primate malaria biology as well at the molecular level.

This Research Topic aims to define malaria at the molecular level in order to gain a better understanding of malaria biology in order to aid in the development of future vaccines. The original article written by Hoque et al. “Identification of Reticulocyte Binding Domain of *Plasmodium ovale curtisi* Duffy Binding Protein (PocDBP) Involved in Reticulocyte Invasion” that describes *P. ovale* DBP function for the first time. The DBP-region II (DBP-RII) is a leading vaccine candidate and an essential domain for invasion of malaria parasite. PocDBP-RII tertiary structure is predicted to be similar to PvDBP-RII which comprises conserved cysteine amino acid residues. Additionally, the function of PocDBP-RII shows binding activity with a specific host cell receptor that could be a Duffy antigen receptor for chemokine (DARC). The original article written by Ahmed et al. “Identification, Mapping, and Genetic Diversity of Novel Conserved Cross-Species Epitopes of RhopH2 in *Plasmodium knowlesi* With *Plasmodium vivax*” characterized high molecular weight rhoptry protein 2 (RhopH2) in clinical samples of *P. knowlesi* and *P. vivax* cross-protective domains, which has been proven to produce cross-protective immune responses in both species (Muh et al., 2020). This study identified a novel conserved RhopH2 epitope in both species and suggests that RhopH2 is a promising vaccine target for protection against both *P. knowlesi* and *P. vivax*. Another study on novel vaccine candidate discovery written by Aparici-Herraiz et al. “Antigen Discovery in Circulating Extracellular Vesicles from *Plasmodium vivax* Patients”, have characterized circulating extracellular vesicles from *P. vivax* patients. The *Plasmodium* secreting proteins are difficult to characterize because of their hyper-variability. Thus, information regarding parasite secreting properties is very limited. This study identified 48 proteins that localize in the vesicle membrane, host cytosol or are exported. This robust identification of exported immunogenic antigens highlight the possibility of significant advances in novel vaccine development strategies.

In *falciparum* malaria, Hou et al. “Merozoite Proteins Discovered by qRT-PCR-Based Transcriptome Screening of *Plasmodium falciparum*” describe blood stage-specific transcriptome of *P. falciparum* and highlight newly discovered proteins that are highly expressed in schizont stage for invasion. This study updates a few missing links of previous knowledge in *P. falciparum* transcriptome information of parasite developmental progression. This section also introduces a novel technique for the characterization of malaria antigen using AGIA tag. The article was written by Morita et al. “AGIA Tag System for Ultrastructural Protein Localization Analysis in Blood-Stage *Plasmodium falciparum*” introduced novel techniques for subcellular protein localization study. The AGIA tag/anti-AGIA rabbit mAb system can be used to elucidate the subcellular localization of understudied proteins in malaria parasites for protein characterization. The last review article written by Molina-Franky et al. “The Cellular and Molecular Interaction Between Erythrocytes and *Plasmodium falciparum* Merozoite” describes the sequential steps of *P. falciparum* merozoite invasion mechanisms at the molecular level. This review focuses in-depth on *P. falciparum* merozoite invasion cascade molecular events and the alterations that occur in a host cell once it has become infected.

Overall, all articles provided novel insight into malaria biology at the molecular level which will aid in the development of future vaccines or therapeutics.

## Author Contributions

All authors contributed to the conception and design of the project. J-HH wrote the first draft of the manuscript. MAA, YC, and FL edited and added sections of the manuscript. All authors contributed to the article and approved the submitted version.

## Funding

This project was partially supported with funding provided by 2020 Research Grant from Kangwon National University (J-HH), National Research Foundation of Korea (NRF) grant funded by the Korea government (MSIT) (NRF-2020R1F1A1071871) (J-HH) and Department of Biotechnology, Govt. of India, No.BT/RLF/Re-entry/09/2017 (MAA).

## Conflict of Interest

The authors declare that the research was conducted in the absence of any commercial or financial relationships that could be construed as a potential conflict of interest.

## Publisher’s Note

All claims expressed in this article are solely those of the authors and do not necessarily represent those of their affiliated organizations, or those of the publisher, the editors and the reviewers. Any product that may be evaluated in this article, or claim that may be made by its manufacturer, is not guaranteed or endorsed by the publisher.
